# SARS-CoV-2 vaccination unmasks distinct immune dysfunctions across lymphoma subtypes and therapies

**DOI:** 10.21203/rs.3.rs-7016519/v1

**Published:** 2025-07-04

**Authors:** Yogambigai Velmurugu, Anna Halling Folkmar Rahimic, Ryan Curtin, Yuan Hao, Samantha Nyovanie, James Langton, Pamela Mishra, Iryna Voloshyna, Akiko Koide, Shohei Koide, Gregg J. Silverman, Ramin Sedaghat Herati, Yury Patskovsky, Catherine Diefenbach, Michelle Krogsgaard

**Affiliations:** NYU school of Medicine; NYU School of Medicine; NYU School of Medicine; NYU School of Medicine; NYU School of Medicine; NYU School of Medicine; NYU School of Medicine; NYU School of Medicine; NYU School of Medicine; NYU School of Medicine; NYU School of Medicine; NYU School of Medicine; NYU School of Medicine; NYU School of Medicine; NYU School of Medicine

**Keywords:** Lymphoma, Hodgkin lymphoma, Non-Hodgkin lymphoma, SARS-CoV-2, cTfh cells, Th1 cells, EMRA T cells, Cytotoxic T cells, anti-CD20 therapy, Chemotherapy Cytotoxic T cells

## Abstract

Patients with lymphoma are at increased risk of severe infections, including SARS-CoV-2, due to immune suppression. Using multidimensional spectral flow cytometry and serology, we characterized in-depth immune responses in 50 SARS-CoV-2 vaccinated lymphoma patients across12 lymphoma subtypes, treated with anti-CD20 antibody (aCD20) ± chemotherapy (CT) or CT alone. Compared to healthy control, aCD20±CT-treated patients exhibited distinct immune alterations, including elevated late-stage effector memory (EM3) CD4+, and terminally differentiated (EMRA) CD8+ T cells, reduced circulating T follicular helper (cTfh) cells, and increased dysfunctional DN3 B cells. While B cell depletion was expected with aCD20 therapy, our data reveals broader immune dysregulation beyond B cell loss. Consistent with these phenotypic changes, aCD20±CT treated patients showed impaired vaccine-induced antibody and T-cell responses. In contrast, CT-only Hodgkin lymphoma patients maintained antibody responses comparable to healthy controls. Notably, SARS-CoV-2-specific T cells in aCD20±CT treated patients displayed fewer regulatory T cells, increased Th1 population, and more EMRA CD8+ T cells, suggesting a compensatory T-cell mediated immunity. Antibody response correlated positively with naïve T cell frequencies and transitional, classical memory, and DN2 B cell subsets. These findings inform the tailored development of vaccine strategies for immunocompromised patients to enhance protection against emerging SARS-CoV-2 variants and other viral pathogens.

## Introduction

The COVID-19 pandemic has provided an unprecedented lens through which to view and deconvolute the complex immunological dysfunction associated with lymphoid malignancies (LM). Patients with lymphoma are inherently immunocompromised due to both their malignancy and its therapies, rendering them particularly vulnerable to severe and prolonged infections. While the efficacy and immunogenicity of COVID-19 vaccines have been studied extensively in cancer populations^1–7^, most prior studies have focused on heterogeneous patient cohorts spanning various cancers and treatment regimens. This heterogeneity in patient population limits the ability to define optimal vaccination strategies for patients with hematological malignancies (HM), especially those receiving immunosuppressive therapies, who remains underrepresented in prior studies. Furthermore, most studies in HM patients have focused primarily on the humoral response to vaccination^1,3,8^ often overlooking the intrinsic immune parameters that modulate vaccine efficacy^9–11^. Given the ongoing evolution and mutation of viruses, understanding the immunological landscape in hematologic malignancy patients is critical for designing novel, effective, and personalized vaccination strategies tailored to this immunocompromised population.

Patients with HM, including classical Hodgkin lymphoma cHL), non-Hodgkin lymphoma (NHL), and lymphoid leukemias, experience higher mortality rates, prolonged hospitalizations, and recurrent COVID-19 infections compared to non-cancer patients and patients with solid tumors^12–19^. A case-control study involving 6,122 vaccinated HM patients reported that 21% developed severe COVID-19^20^. In multiple myeloma (MM), the risk of breakthrough infections was 15.4%, markedly higher than the 3.9% observed in the general non-cancer population^21^. While SARS-CoV-2 vaccination did not significantly reduce the risk of mortality in HM patients, it was associated with a reduction in hospitalization rates^22^, perhaps suggesting that it improved outcomes for patients able to mount some antiviral immune response, but not for the most immune deficient.

Severe COVID-19 outcomes in lymphoma patients are largely driven by disease-associated immune dysregulation and immunosuppressive therapies such as anti-CD20 (aCD20) antibodies that target and deplete B cells^23,24^. However, studies have shown that HM patients with impaired T cell responses, regardless of B cell activity experienced the highest mortality rates^24^, suggesting that a humoral response alone is insufficient for protection from acute COVID-19 infection. Notably vaccine-induced CD8 T cell responses to SARS-CoV-2 spike protein epitopes were comparable between lymphoma patients and healthy controls^23^, and elevated circulating CD8 + T cell counts were associated with survival in HM patients following COVID-19 infection^24^. Despite these findings, a systematic analysis of the vaccine-induced T cell subpopulations or their relationship with global (bulk) T and B cell phenotypes has not been described in lymphoma patients, particular in the context of attenuated SARS-CoV-2-immunity.

Our study provides a comprehensive analysis of SARS-CoV-2 vaccine-induced immunity in lymphoma patients undergoing different treatments, including anti-CD20 antibody (aCD20) therapy, chemotherapy (CT), or both in combination (aCD20 + CT), as well as patients with indolent B cell lymphoma monitored expectantly without active treatment (MON). Detailed phenotypic profiling of global T and B cell populations in aCD20 ± CT-treated patients revealed significant alterations in lymphocyte subsets, including increased late-stage effector memory (EM3) CD4 + and terminally differentiated (EMRA) CD8 + T cells, reduced T follicular helper (cTfh) cells, and elevated dysfunctional DN3 B cells compared to healthy controls. These changes highlight immune dysregulation beyond direct B cell depletion by aCD20. Our findings further indicate that while vaccine-induced cellular immunity is moderately affected by cancer treatments, humoral immunity is more profoundly influenced by disease-state and therapy type. Importantly, CT-treated Hodgkin lymphoma (HL) patients, with reduced B cell percentages but with intact CD4 + T cells phenotypes, still able to retain antibody responses, suggesting a critical role for CD4 + T cells in supporting antiviral humoral immunity.

Whereas we observed weakened humoral immunity in aCD20 treated lymphoma patients, we also detected a concurrent decrease in T regulatory cells and an increase in Th1 cells and EMRA CD8 + T cells within the vaccine-induced, SARS-CoV-2-specific T cell population. A compensatory increase in CD8 + T cell response may rationalize these findings^24,25^. Overall, our data suggests that optimizing vaccination strategies for viral infections in lymphoma patients may require additional vaccines or novel vaccine regimens designed to specifically elicit specific targeted immune mediators, thereby promoting faster and more effective protection against SARS-CoV-2 and other pathogens.

## Results

### Cancer therapy type determines humoral immunity in lymphoma patients.

**Figure 1:**
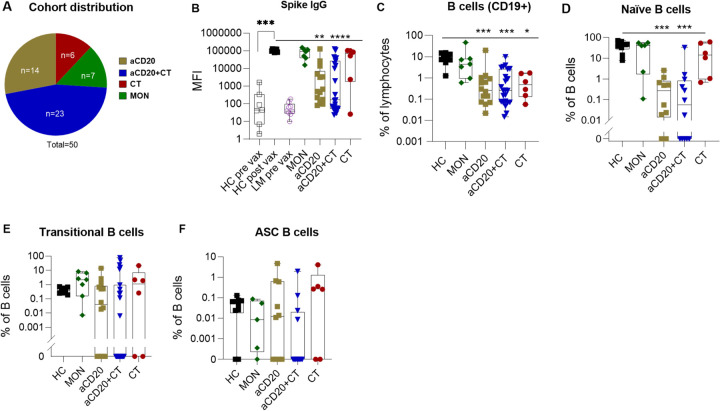
SARS-CoV-2 vaccine-induced antibody response and B cell subsets vary by type of lymphoma treatment (A) Pie chart showing the number of patients in each lymphoma subtype included in this study. (B) anti-spike IgG levels in lymphoma (LM) patients and healthy controls (HC), post second vaccination. (C-F) frequencies of B cell subsets of post vaccinated mononuclear leukocytes (PBMC’s) of HC and LM patients under different treatments (patients under monitoring (MON), anti-CD20 therapy (aCD20), combination therapy with both anti-CD20 and chemotherapy (aCD20+CT), chemotherapy alone (CT)) using traditional gating strategy (representative flow cytometry plots of the gating strategy for the B cells is shown in Extended Fig.1D). In figures C, D, E, F each dot represents individuals in HC or LM cohorts. Data are shown as mean value +/−SEM. Statistical comparisons between healthy and LM cohorts were performed using Kruskal-Wallistest then Dunn’s test was applied as a post-hoc test for multiple comparison. *p<0.05; **p<0.01; ****p<0.0001; ns not significant.

All human samples were collected on an IRB-approved protocol between March 2021 to July 2021. Fifty patients were recruited who were diagnosed with 11 types of lymphoma: Follicular Lymphoma fl, Marginal Zone Lymphoma [MZL], Diffuse Large B-cell lymphoma [DLBCL], classical Hodgkin Lymphoma [cHL], lymphocyte predominant Hodgkin lymphoma (LPHL), Waldenström Macroglobulinemia [WM], Chronic Lymphocytic Leukemia [CLL], Burkitt lymphoma [BL], low-grade lymphoma (LG), Mantle cell lymphoma(MCL) and Mycosis Fungoides [MF]), who received one of four different treatment regimens (Mon, aCD20, aCD20 + CT, CT). These enrolled patients were either on treatment at the time of vaccination or, had completed treatment within 6 months (Fig. 1A, Extended Fig. 1A, Table 1).

We used a custom multiplex bead immunoassay^26^ designed for the high-sensitivity detection of antibody responses to SARS-CoV-2 induced by vaccination. Compared to their pre-vaccination levels, we observed a significant increase in vaccine-induced anti-spike IgG levels among vaccinated lymphoma (LM) patients (p = 0.0001) (Extended Fig. 1B). However, comparisons of post-vaccination antibody levels among lymphoma patients who received various treatments to healthy control (HC) revealed variable responses, indicating the influence of the treatment. Notably, only 45% of lymphoma patients receiving aCD20 ± CT therapy exhibited an antibody response to the vaccine (Fig. 1B), with the lowest response in aCD20 + CT, compared to HC (p < 0.00001). In contrast, 83% of lymphoma patients, primarily those with cHL who received CT alone (80% of cHL, 10% MZL, and 10% WM), exhibited a robust antibody response, with four out of six having antibody levels comparable to HC (Fig. 1B and Extended Fig. 1A). Our previous studies^27^ indicated that all cHL patients treated with CT mounted an antibody response, albeit diminished compared to HC suggesting that in cHL patients chemotherapy preserves humoral responses, which contrasted with those who had received B cell-directed therapy. When evaluated by specific cancer type, FL and DLBCL patients showed significantly diminished IgG response to vaccination compared to the HC cohort (p = 0.0063, p < 0.00001) (Extended Data Fig. 1C). All FL and DLBCL patients, except one, underwent aCD20 ± CT therapy, suggesting that aCD20 treatment was a key determinant in their lack of antibody response. These results concur with previous data demonstrating that patients with hematologic cancer treated with aCD20 exhibited the lowest levels of vaccine-induced neutralizing antibodies compared to all other treatments combined, including CT^1^. Remarkably Waldenstrom’s macroglobulinemia patients showed comparable antibody response to HC irrespective of their treatments (CT-1, aCD20 + CT-1, aCD20–1, MON-1). All patients in the Mon group, primarily MZL patients (43%), showed induced anti-spike IgG responses that were comparable to HC. However, our earlier study found that only 73% of CLL patients in the Mon group had this humoral response^27^, suggesting that other cancer disease-related factors may influence the immune response to vaccination. Furthermore, while aCD20 treatment significantly impairs humoral responses in lymphoma patients, antibody responses also vary by cancer subtype.

### The presence of specific B-cell phenotypic subsets correlates with SARS-CoV-2-induced antibody production, even in patients undergoing B-cell targeted therapy.

To explore how anti-CD20 treatment affects immune features and how the representation of B cell phenotypic subsets correlates with vaccine-induced antibody production, we utilized high-dimensional flow cytometry,^28^ to compare the levels of peripheral B lymphocyte subsets in post-vaccinated lymphoma patients and HC (Extended Tables 2 and 3, Extended Fig. 1D). We observed a significantly lower frequency of CD19^+^ B cells across all groups of NHL and HL patients including those receiving aCD20 ± CT, or CT alone (p = 0.0003, p = 0.0001 and p = 0.0157 respectively) compared to HC (Fig. 1C). Consistent with earlier reports^29–32^, we found that B cell depletion therapy diminishes overall B cell populations, with CT having a similar effect. Notably, in contrast, untreated NHL patients (MON) exhibited a B cell frequency similar to HC. In general, we found a positive correlation between anti-spike antibody responses and the proportion of B cells within the total circulating lymphocytes in lymphoma patients (r = 0.45, p = 0.008, Extended Fig. 1E), which suggested that B cells that repopulate the circulation pool after depletion therapies remain functionally competent, akin to a previous study in multiple sclerosis receiving aCD20 treatment^25^.

**Figure 2:**
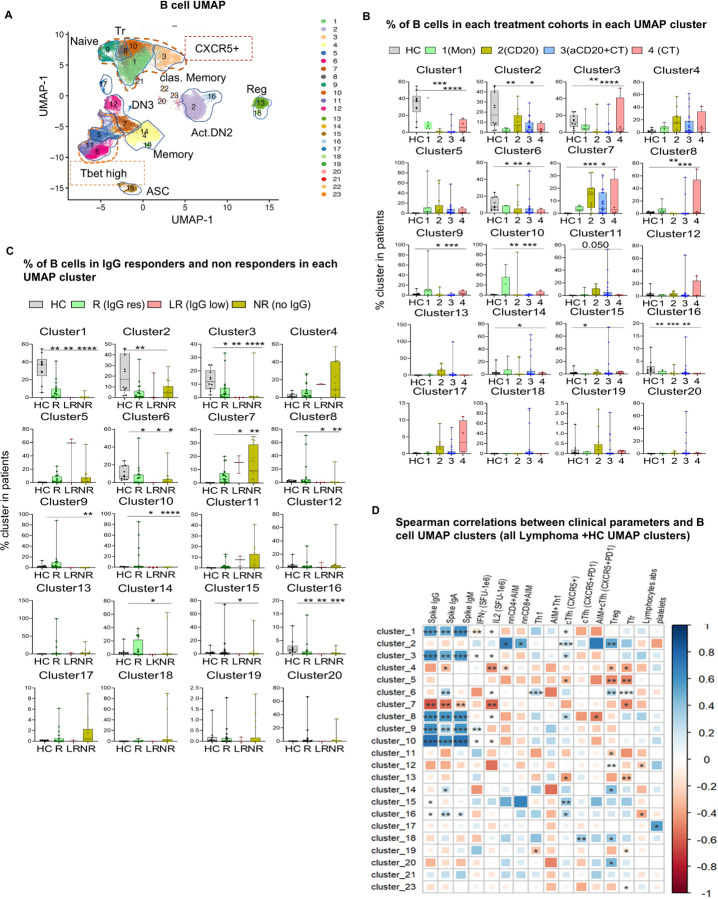
SARS-CoV-2 vaccine induced antibody response correlated with certain B cell subsets. (A) UMAP visualization of HC and lymphoma patientsanalyzed together (LM and HC) and colored by manually annotated cell types; DN1,2,3- double negative B cells, naïve B cells, Reg- regulatory B cells, ASC-antibody secreting B cells. (B, C) Bar plots of the percentage of B cells from each cohort in each UMAP cluster; cohorts were defined by (B) treatment types, or (C) by antibody levels (responders (R): IgG>1000 a.u., non-responders (NR): IgG<399 a.u., and low responders (LR): IgG levels between 400–1000 a.u.). In figures A and B, each dot represents individuals in HC or LM cohorts. (D) Spearman correlations of clinical parameters and antibody responses with B cell UMAP cluster frequencies (both healthy and LM clusters analyzed together). Detailed phenotypes of B cell clusters listed in extended table 4. Each dot represents individuals in HC or LM cohorts. Data are shown as mean value +/−SEM. Statistical comparisons between healthy and LM cohorts were performed using Kruskal-Wallis test then Dunn’s test was applied as a post-hoc test for multiple comparison. *p<0.05; **p<0.01; ****p<0.0001; ns not significant.

Treatment with aCD20 ± CT significantly reduced the percentage of naïve B cells compared to HC, attributable to treatment- and disease-related immune deficiency (p = 0.0001, p = 0.0008 respectively) (Fig. 1D). In contrast, treatment groups that exhibited antibody response comparable to HC (MON, CT) displayed similar or elevated naïve, transitional (Tr), and antibody-secreting (ASC) B cell populations, highlighting the potential contribution of these B cell subtypes to immune functionality (Fig. 1D, E, F). Because traditional gating methods were challenged by low B cell numbers in cancer patients, to analyze B cell phenotypes and compare B-cell phenotype frequency across treatments, we employed uniform manifold approximation and projection (UMAP) visualization with unsupervised clustering^33,34^ (Fig. 2A, Extended Fig. 2A, B, Table 4). In patients treated with aCD20 ± CT, compared to HC we observed significant reductions in Tr (clusters 8) and naïve (clusters 1, 9 and 10) cell frequencies, which corroborated results demonstrated with our traditional gating strategy (Fig. 1D, E, Fig. 2A, B). Patients in the MON and the CT groups had similar frequencies to HC in their naïve and Tr clusters. Notably, patients with higher level serum antibody responses (IgG > 1000 a.u.) possessed greater representation of naïve and Tr clusters compared to non-responders, even in patients with severe overall B cell depletion (Fig. 1C, Fig. 2C). We observed strong correlations between levels of spike-specific antibody responses with Tr and naïve B cell populations (Fig. 1D: correlation between UMAP clusters 8, 1,9, and 10 with anti-spike IgG (both healthy and lymphoma patients combined) (r = 0.6, p < 0.0001; r = 0.6, p < 0.0001; r = 0.6, p < 0.0001; r = 0.8, p < 0.0001 respectively), and extended Fig. 2C–E: Tr, naïve and ASC B cell subpopulation of lymphoma patients from traditional gating correlates with spike IgG). These findings indicate that, despite reductions in total peripheral B cell number patients retaining both transitional and naïve B cell percentages within the B cell population retained the capacity to generate SARS-CoV-2-specific antibody responses. Further, in aCD20 ± CT-treated patients, resting class-switched memory B cells (classical memory), represented by cluster 3, were significantly decreased compared to HC (aCD20, p = 0.002 and aCD20 + CT, p < 0.0001) (Fig. 2A, B). Correspondingly, patients exhibiting strong IgG anti-spike responses had a higher frequency of classical memory B cells (cluster 3) compared to non-responders, and the representation of cluster 3 strongly correlated with the magnitude of the IgG antibody responses (r = 0.6, p < 0.0001) (Fig. 2C, D). Our findings suggest that lymphoma patients with frequencies of classical memory B cell populations comparable to HC may retain functionally competent B cell populations capable of mounting effective responses to vaccination.

Next we examined atypical CD19 + IgD− CD27-B cells, termed double-negative (DN), which have been implicated in the pathogenesis of a range of diseases, including responses to COVID-19 infection, certain cancers, chronic inflammatory disorders, and autoimmune diseases^35^. DN B cells comprise several heterogeneous subgroups (DN1, DN2, DN3) with distinct functions^8,28,36–39^. Prior studies have identified DN2 cells as plasma cell precursors that can undergo further differentiation through an extra-follicular pathway^40,41^. Recent findings have further demonstrated that in response to SARS-CoV-2 vaccination, DN2 B cells promptly expand and are pre-primed for differentiation into ASCs^42^. In our studies, higher levels of this activated DN2 subset (clusters 2,16, CD11C + CD21−HLA-DR + CD40+) were found in HC than in lymphoma patients (Fig. 2A, B). The activated DN2 population in clusters 2 and 16 weakly correlated with the level of cTfh within CD4 T cells and with total antigen-specific CD4 and CD8 T cell populations (Fig. 2D). Additionally, the DN2 cell cluster 16 (IgG+, class switched) moderately correlated (r = 0.3, p = 0.03) with anti-spike antibody levels (Fig. 2D), suggesting extrafollicular differentiation of DN2 cells into ASCs with cTfh cell help^35,43^. Moreover, lymphoma patients had slightly higher frequency of DN2 cells in clusters 11 and 5 (Tbet high, CD11C-CD21-) compared to HC. Despite reductions in total peripheral B cell numbers, frequency of B cells in DN3 cluster 7 (CD11C− CD21− Tbet−) is significantly high both in the monitoring and treatment groups, compared to HC, reflecting increased DN3 cells in cancer patients, as previously reported^39,43,44^ (Fig. 2A, B). The highest levels of the DN3 population (cluster 7) were observed in aCD20 ± CT-treated patients (p = 0.017 and p = 0.0008, respectively). Notably, the DN3 cluster 7 frequency, negatively correlated with serum anti-COVID-19 antibody levels (r=−0.6, p = 0.0024) (Fig. 2D).

In summary, these findings suggest that the frequencies of certain B cell subsets, including Tr, naïve, classical memory, and DN2, predict SARS-CoV-2 vaccine-induced antibody responses, even in patients with reduced total circulating B cell frequency following aCD20 treatment.

### Most lymphoma patients exhibit SARS-CoV-2-specific T cell responses comparable to healthy controls.

A recent meta-analysis of 17 publications found that approximately two-thirds of hematologic cancer patients developed T cell responses after SARS-CoV-2 vaccination, although the degree of response varied widely depending on cancer type and treatment^45^.

**Figure 3:**
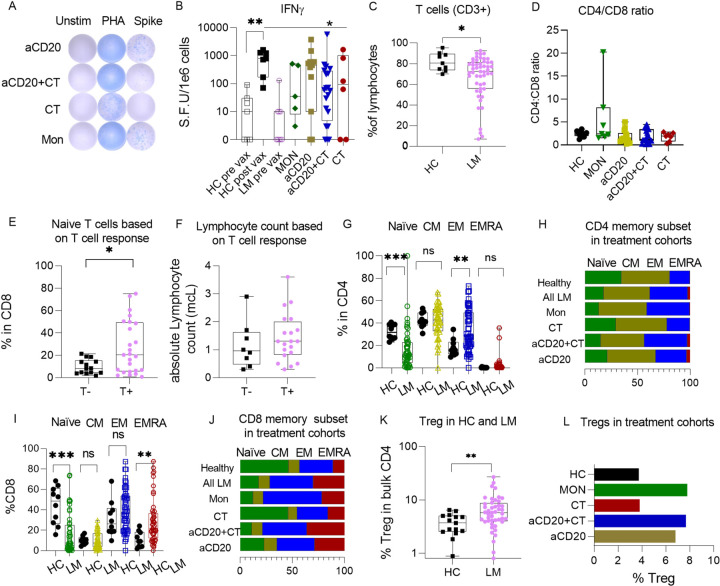
SARS-CoV-2 vaccine-specific T cell responses in lymphoma patients are linked to distinct T cell subset. (A) Representative cytokine ELISpot assay responses of lymphoma (LM) patients under various treatments against SARS-CoV-2 antigen with DMSO as a negative control (unstim) and PHA as a positive control. (B) IFN-gresponse to the SARS-CoV-2 spike peptide library stimulation measured by ELISpot assay. (C-F) Comparison of T cell phenotypes in PBMC’s (post vaccinated) between LM and HC. (C) Comparison of CD3+T. (D) CD4/CD8 ratio. (E) comparison of naïve T cell in T cell responders (T+) and non-responders (T−) as measured by ELIspot(T+>18 SFU per million cells and T− <18 SFU per million cells). (F) Absolute lymphocyte counts between T cell responders (T+) and non-responders (T−). (G-J) Comparison of (G) CD4+naïve, CM, EM, and EMRA cells in LM patients analyzed together and HC. (H) CD4+ naïve, CM, EM and EMRA frequencies of LM patients under various treatments and HC. (I) CD8+naïve, CM, EM, and EMRA cells in LM patients analyzed together and HC. (J) CD8+ naïve, CM, EM, and EMRA frequenciesin LM patients under various treatments and HC. (K) Variation in T regulatory cells between LM patients and HC. (L) Comparison of T regulatory cells in LM patients under various treatments. Representative flow cytometry plots of the gating strategy for the naïve, CM, EM, and EMRA cells are shown in extended Fig.7(E). In Figures (B, C,D,E,F,G, I, K), each dot represents an individual either in HC or in different LM cohorts.Data were shown with mean+/−SEM. Statistical comparison between two data set were performed with the Mann-Whitney test. The Kruskal-Wallistest was used for multiple comparison across treatment groups and HC then Dunn’s test was applied as a post-hoc test for multiple comparison. *p<0.05; **p<0.01; ****p<0.0001; ns not significant.

In our study, we examined how lymphoma histologic subtypes and different treatments influence post-vaccine T cell responses. To assess IFN-γ release during T cell response to antigen, we used an enzyme-linked immunospot assay (ELISpot) after the stimulation of PBMCs with spike protein peptides from the SARS-CoV-2 Delta variant (Fig. 3A, Extended Fig. 3A). Compared to pre-vaccination levels, after vaccination lymphoma patients displayed a significant increase in the frequencies of spike-specific IFN-γ-secreting T cells (p = 0.0028) (Extended Fig. 3B). Despite great variability in the lymphoma group, the mean fold change in IFN-γ production between pre- and post-vaccination was comparable to HC, as the mean change between pre- and post-spot forming unit (SFU) per million cells in HC was 38.4-fold (p = 0.0012), and in lymphoma 37.8-fold (p = 0.0028) (Extended Fig. 3B). However, only 72% of the lymphoma patients attained levels of SFU counts that were comparable to those in HC (Fig. 3B). Notably, patients treated with aCD20 + CT exhibited significantly lower SFU counts compared to the HC (p = 0.023) (Fig. 3B).

Previous studies of patients with COVID-19 infection have indicated that those with severe diseases generally subsequently develop stronger T cell responses against SARS-CoV-2^46^. In our study, compared to HC across all cancer types, there was a trend toward lower T cell responses; however, this decrease did not reach statistical significance (Extended Fig. 3C). Despite evidence that vaccination enhances protection against adverse outcomes associated with severe COVID-19 infection^11^, lymphoma patients continue to face a higher risk of severe infection compared to HC.

### Treatment-dependent alterations in naïve, memory, and activated T cell phenotypes in NHL and HL patients

Lymphoma biology and treatment directly impact immune system function. Previous studies demonstrated abnormalities in the T-cell populations of hematologic cancer patients^47–49^. In addition, SARS-CoV-2 disease severity has been correlated with lymphopenia, especially in the elderly or in cancer patients^50–55^. We therefore aimed to determine if impaired SARS-CoV-2-specific T cell priming in lymphoma patients was associated with T cell defects caused by cancer or with its treatment.

The frequency of CD3 + T cells in lymphocytes and CD4 + and CD8 + subsets within CD3 + T cells varied between lymphoma patients (Fig. 3C, Extended Fig. 3D, E). Despite higher inter-patient variability, lymphoma patients did not show a significant decrease in CD4 + or CD8 + T cell frequencies compared to HC (Extended Fig. 3D, E). However, compared to HC, patients in the monitoring group displayed a trend toward a higher CD4+/CD8 + ratio, a hallmark of lymphoma (Fig. 3D)^56^. Only 60% of vaccinated patients in the MON group produced a vaccine-induced T cell response that was comparable to HC. Studies of patients with acute COVID-19 infection have found a correlation between the representation of the naïve T cell population in the circulation and the magnitude of the T cell response^57^. Likewise, a paucity of naïve T cells is a risk factor for severe COVID-19 disease^58,59^. Consistent with previous findings^60^, lymphoma patients who mounted a T cell response had a significantly higher proportion of naïve CD8 + T cells compared to non-responders (p = 0.0379). Moreover, aCD20 + CT treated patients exhibited the lowest representation of naïve T cells, indicating that both lymphoma subtype and treatment are associated with reductions in naïve T cell populations (Fig. 3E, Extended Fig. 3F, G). Compared to non-responders, the total lymphocyte counts in PBMCs were higher in T cell responders (Fig. 2F).

**Figure 4:**
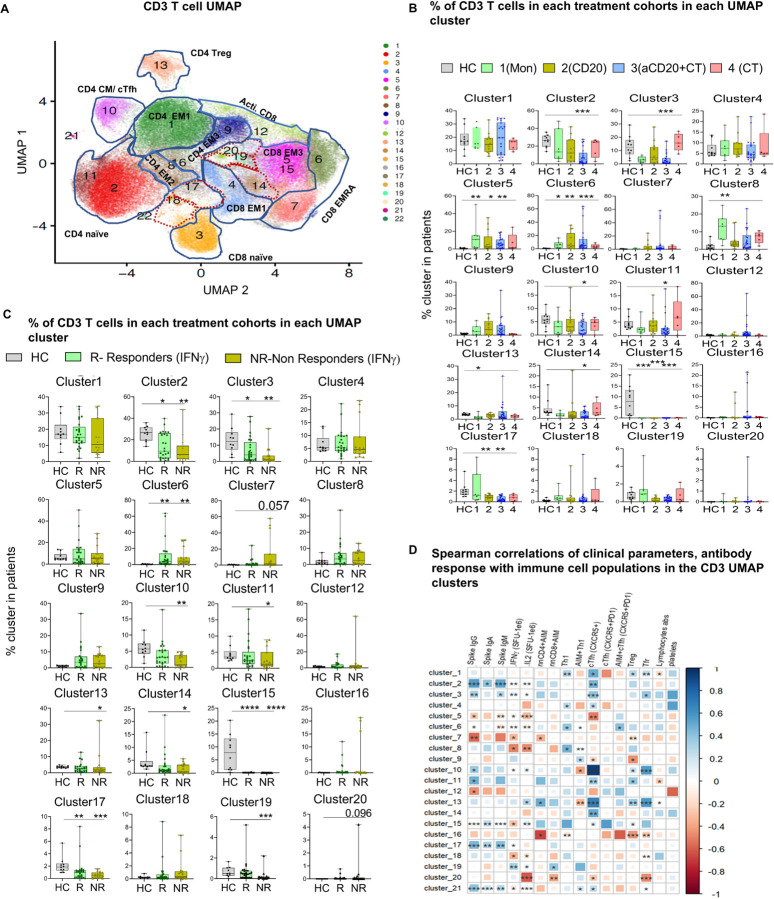
SARS-CoV-2 vaccine-specific T cell responses in lymphoma patients correlate to distinct T cell subset (A) UMAP representation of CD3 T cells in PBMCs with all samples analyzed together (LM and HC) and colored manually annotated cell phenotypes. T cell subgroups were identified in CD4+ T cell and CD8+ T cell clusters (Naïve, CM, EM1, EM2, EM3, EMRA, regulatory (Treg), Th1, cTfh as indicated in the figure). Red contour maps include SARS-CoV-2 antigen-specific(AIM+) T cells. (B, C) Bar plots of the percentage of T cells from each cohort in each UMAP cluster. (B) Cohorts were defined by treatment types. (C) Cohorts were defined by ELISpot responders (T+:R) and non-responders (T−: NR). Patients not analyzed by ELISpot assay was omitted in the histogram. In figures (C and D), each dot represents an individual either in HC or in different LM cohorts. (K) Spearman correlations of clinical parameters and antibody responses with T cell UMAP clusters. The Kruskal-Wallistest was used for multiple comparison across treatment groups and HC then Dunn’s test was applied as a post-hoc test for multiple comparison. *p<0.05; **p<0.01; ****p<0.0001; ns not significant.

To further dissect the complexity of the T cell response, we quantified the memory T cell subsets (T_CM_, T_EM_, T_EMRA_), regulatory T cells (Treg), and helper T cells (Th1, cTfh) within both CD4 + and CD8 + T cell compartments, using conventional flow cytometry gating strategies alongside independent UMAP visualization^61^ (Fig. 3G–L, Fig. 4A, extended Fig. 4A, B). To identify biomarkers predictive of vaccine response, cluster frequencies were then correlated with clinical key features^62^ (Fig. 4A–D). The traditional gating analysis revealed that lymphoma patients had a lower percentage of naïve cells across all groups except CT. Conversely, lymphoma patients also showed a higher percentage of differentiated T cells, particularly CD4 EM and CD8 + EMRA cells (Fig. 3G–J). The reduction in the representation of naïve T cells was mainly due to the enrichment of antigen-experienced memory T cells^49,63–65^, which was especially pronounced (CD4 + naïve, p = 0.0007, CD8 + naïve = 0.0004 (Fig. 3G, I). In cancer patients, the presence of T_CM_ and T_EM_ cells has consistently been associated with better prognosis^24,49,66^. When comparing different lymphoma treatment groups, memory T cell percentages in the CT cohort were comparable to HC (Fig. 3H, J). UMAP analysis identified 25 clusters (Supplementary Table 5), and consistent with traditional gating, lymphoma patients showed reduced frequencies of naive CD4 + and CD8 + T cells (clusters 2,11, and 3) (Fig. 4A, B). In both lymphoma patients and HC, unsupervised cluster analysis showed that levels of naïve T cell populations correlated with the level of induced antibody responses (cluster 2, r = 0.6, p < 0.0001, cluster 11 (CD38+, ICOS+), r = 0.5, p = 0.01, and cluster 3, r = 0.3, p = 0.007) (Fig. 4D). In lymphoma patients, traditional gating analysis also revealed a moderate direct correlation between the representation of naïve T cells and the magnitude of anti-spike IgG, and IFNg (Extended Fig. 5A, B, C, D). By cluster analysis, the EM subsets were further divided, revealing that CD4 + EM2 T cells (cluster 8), which express CD25, were enriched in lymphoma patients, particularly in the MON group (p = 0.0019). The frequency of this cluster negatively correlated with IFNg production following in vitro SARS-COV-2 antigen stimulation (r=−0.5, p = 0.04) (Fig. 4A, B, and D). CD4 + EM3 T cells (cluster 9), expressing ICOS and PD-1, were enriched in lymphoma patients, except those who had received CT alone (Fig. 4A, B). Our correlation analysis indicates that patients with a higher proportion of circulating naïve cells were more likely to mount a positive T cell response to vaccination. In contrast, a greater abundance of late-stage differentiated effector memory cells was associated with a poorer T cell response.

In aCD20 + CT treated patients, activated CD8 + EM3 cells (cluster 12), which express HLA-DR and T-bet^67^ (cluster 12), were enriched (Fig. 4A, B), and their negative frequency correlating with anti-spike IgG responses (r= −0.6, p = 0.005) (Fig. 4D). Compared to HC, lymphoma patients -except those treated with CT alone – showed higher representation of more differentiated cytotoxic CD8 + EMRA subsets, expressing CD38 (cluster 6, 7). Specifically, cluster 6 frequencies were elevated in MON (p = 0.0205), aCD20 (p = 0.009), aCD20 + CT (p = 0.0005) (Fig. 4A, B), and these frequencies negatively correlated with antibody response (r=−0.5, p = 0.05) (Fig. 4D).

CD4 + T follicular helper cells (cTfh), which assist B cells within the germinal center, are essential for generating a strong antibody response,^68^ were reduced in lymphoma patients compared to HC. This reduction was even more pronounced in the aCD20 + CT group (cluster 10, p = 0.017) (Fig. 4A, B, Extended Fig. 5E). We speculate that this decrease may partly result from B cell depletion-related dysregulation of cTfh in these patients. Furthermore, the frequency of cTfh populations correlated with the level of IgG antibody responses (r = 0.4, p = 0.04) (Fig. 4D, Extended Fig. 5F). Lymphoma patients also exhibited greater heterogeneity in the peripheral representation of activated cTfh (ICOS + CD38+)^68,69^ (Extended Fig. 5G). In contrast, the Th1 subset, which enhances cytotoxic T-cell responses during viral clearance,^70^ showed comparable frequencies between lymphoma patients and HC (Extended Fig. 5H).

Principal components analysis (PCA) of CD3 + cell clusters showed that lymphoma patients and HC occupy non-overlapping PCA space, reflecting great differences in the representation of defined T cell subsets between lymphoma patients and HC (Extended Fig. 6A). Compared to lymphoma patients, the HC exhibited less intragroup variation in T cell profiles. Furthermore, the greatest heterogeneity was found in patients treated with aCD20 ± CT, whereas CT patients were closer to HC, reflecting NHL/HL subtypes and varying treatment effects.

In summary, NHL and cHL patients receiving various treatments exhibited substantial heterogeneity of the CD3 + peripheral compartment, with the NHL aCD20 + CT group showing the most pronounced differences compared to HC. This may reflect the loss of costimulatory signals and cytokine-mediated regulation normally provided by B cells to CD4 + T cell subsets. Despite the altered T cell phenotypes in LM patients treated with aCD20 + CT, these individuals displayed an increased proportion of late stage activated CD8+ T cells^71^, likely indicative of heightened T cell activation in response to ongoing disease.

### The interplay between T and B cells coordinating effective humoral and cellular response to SARS-CoV-2 mRNA vaccination.

The bidirectional interactions between B cells and T cells play critical roles in effective vaccine responses. While overall anti-spike IgG responses weakly correlated with IFN-γ (r = 0.3, p = 0.04) (Extended Fig. 6B), certain treatment groups, such as CT and MON, exhibited better correlations (r = 0.4, p = ns, and r = 0.8, p = ns, respectively), emphasizing the importance of efficient crosstalk between B and T cells. The majority of DLBCL patients treated with aCD20 + CT expressed neither IgG or IFN-γ (Fig. 1B, Fig. 3B, Extended Fig. 1C, and Fig. 3C). Additionally, specific T cell clusters, including naïve CD4 + and CD8+, naive CD4+(CD38 + ICOS+) and cTfh cells, correlated with certain B cell clusters such as naïve, Tr, DN1, DN2, and ASC (Extended Fig. 6C).

### Effect of age on humoral and cellular immune responses

Lymphoma patients were older than the HC (mean age: 62 vs. 42, respectively) (Extended Fig. 6D). Age correlated negatively with levels of naïve CD4 + and CD8 + T cells in lymphoma patients (r=−0.34, p = 0.0153, r=−0.59, p < 0.0001) (Extended Fig. 6E, F). However, age had little impact on IFN-γ or IgG antibody production in lymphoma patients, with cancer type and treatment playing a more substantial role in immune response (Extended Fig. 6G).

### Lymphoma patients elicited SARS-CoV-2 vaccine-specific memory T cell responses comparable to healthy controls.

**Figure 5:**
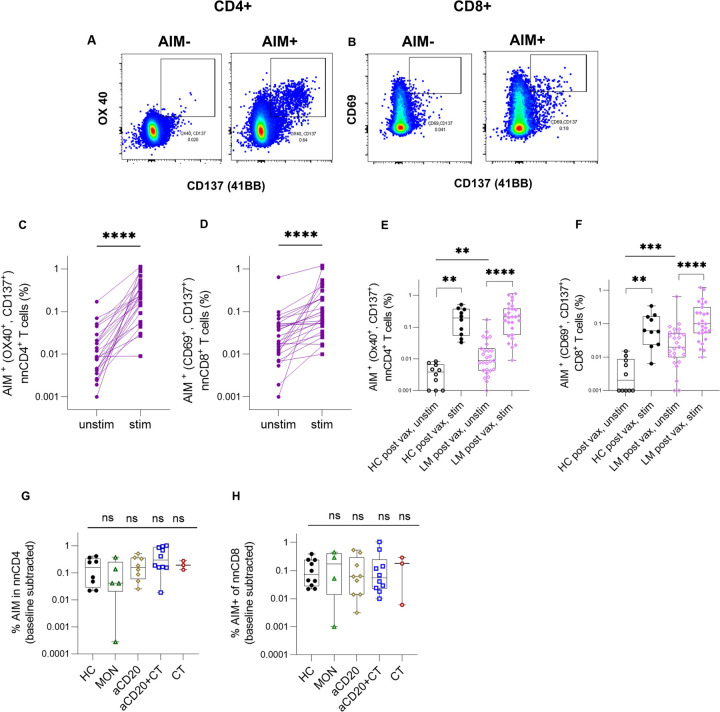


SARS-CoV-2 vaccination induces specific memory T cells, crucial for rapid immune responses upon re-exposure to the virus, which contributes to protective immunity against COVID-19^72^. After stimulating PBMCs with a spike-specific peptide library, SARS-CoV-2-specific CD4 + T cells were identified through their dual expression of the activation markers OX40 and 41BB, whereas CD8 + T cells were identified by dual expression of CD69 and 41BB^57,73–77^ (Fig. 5A, B). The activation-induced marker (AIM) analysis of PBMCs showed results similar to the ELISpot assay (Fig. 3A, B). When identifying AIM + T cells in responders, we included only 25 lymphoma patients due to low PBMC counts in the remaining patients. Among these 25 lymphoma patients, 23 and 21 had detectable CD4 + and CD8 + memory T cell responses, respectively (Fig. 5C, D). After vaccination, both NHL/HL patients and HC exhibited similar frequencies of antigen-specific T cells (Fig. 5E, F). These findings indicate that lymphoma patients who mounted T cell responses exhibited vaccine-induced T cells that were comparable to those of HC. In contrast, pre-pandemic PBMCs from SARS-CoV-2 vaccine-naïve lymphoma patients and HCs showed no significant memory T cell responses upon stimulation (Extended Fig. 6A, B).

Interestingly, lymphoma patients exhibited higher baseline levels (unstimulated) of AIM marker expression in CD4 + and CD8 + T cells compared to HC post-vaccination (CD4, p = 0.0096 and CD8, p = 0.0002), suggesting preexisting low-level immune activation^71^ potentially due to malignancy-related inflammation (Fig. 5E, F). No significant differences in the representation of antigen-specific T cells were observed between different treatment types (Fig. 5G, H). In lymphoma patients, a weak correlation was noted between the frequency of antigen-specific memory T cells and naïve T cells (CD4, r = 0.14, p = 0.38 (ns) and CD8, r = 0.31, p = 0.047) (Extended Fig. 6C, D) indicating that the abundance of naïve T cells is critical for mounting virus-specific responses^74^.

In summary, the magnitude of SARS-CoV-2-specific memory T cells capable of mounting IFNg responses in lymphoma patients was not differentially influenced by the type of cancer treatment. We hypothesize that lymphoma patients may compensate for altered B and T cell populations through an increase in certain antigen-specific T cell phenotype, compared to healthy controls. Further immune profiling is warranted to elucidate these potential compensatory mechanisms.

### Lymphoma patients show increased EMRA cells and decreased CM cells in the vaccine-induced antigen-specific population.

**Figure 6:**
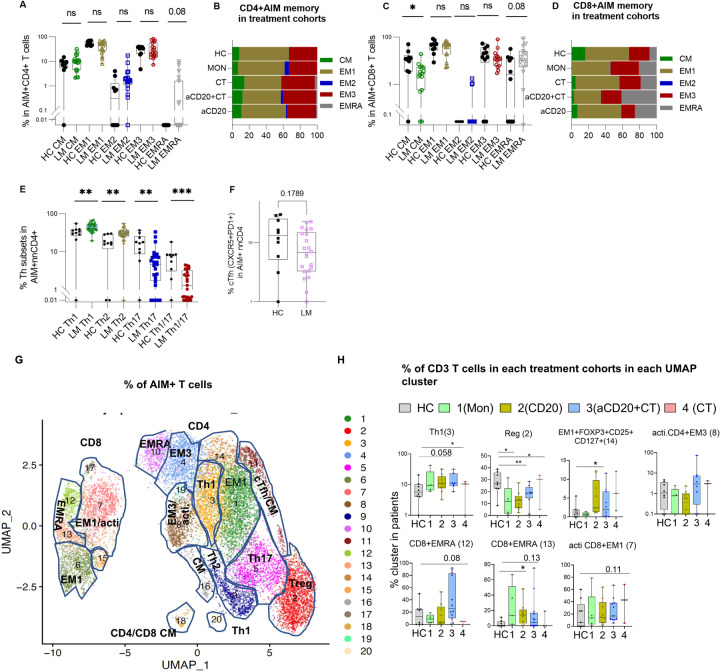
SARS-CoV-2 mRNA vaccination-induced memory T Cell phenotype in lymphoma patients but with alteredTh1, regulatory and EMRA CD8+ T cell populations compared to HC. (A-D) Comparison of the frequency of antigen-specific memory T cell subsets in lymphoma patients and healthy controls (A,B) AIM+CD4+T cells, and (C,D) AIM+CD8+ T cells. Representative flow cytometric plots depicting the gating of AIM+CD4+ and AIM+CD8+ T cells to identify the memory T cells subsets in post vaccinated samples is shown in the extended Fig. 7 E and extended Fig.8 A,B: Red events depict AIM+CD4+ T cells, and gray events depict total CD4+ T cells. Boxplots represent the median with an interquartile range. (E) Comparison of the frequencies of T helper subsets (Th1,2 and 17) in AIM+ CD4+T cells in LM patients and HC. (F) Comparison of the frequencies of circulating follicular helper subsets (cTfh) in AIM+CD4+ T cells in LM patients and HC. cTfh: CXCR5+ nnCD4+ T cells; Th1: CXCR5-CXCR3+CCR6-CCR4−; Th2: CXCR5-CXCR3-CCR6-CCR4+; Th17: CXCR5-CXCR3-CCR6+CCR4-. (G) UMAP representation of AIM+CD4 and AIM+CD8+ T cells in PBMCs from all samples analyzed (LM and HC) and colored by manually annotated cell types. T cell subgroups were identified in AIM+CD4+ T cell and AIM+CD8+Tcell clusters (CM, EM1, EM2, EM3, EMRA, regulatory (Treg), Th1, cTfhas indicated, act. represents activated cell population within the antigen specific cells (H) Bar plots of the percentage of T cells from individuals in each treatment cohort in each UMAP clusters. All statistics were calculated using Mann-Whitney test.When plotting cluster frequencies in Fig.6.H, patients with AIM+CD4+ T cells counts less than 15 cells were excluded from the analysis. For AIM+CD8+ T cells cluster frequency plots no such restriction in cell counts were imposed due to overall low cell counts in AIM+CD8+T cells.

To assess vaccine-specific memory T cell differentiation, we examined CM, EM types EM1, EM2, and EM3, and EMRA T cell populations. These subsets, distinguished by expression of CD45RA, CD27, and CCR7^78,79 62,80,81^, exhibit distinct homing and effector properties (Extended Fig. 7E). Post-vaccination, both lymphoma patients and HCs displayed elevated long-lived EM1 memory^82^ populations in antigen-specific T cells compared to EM1 in bulk T cells (Extended Fig. 7F, G). However, lymphoma patients had fewer CD8 + CM cells compared to HC (p = 0.014) and slightly more CD8 + EMRA within the antigen-specific population (Fig. 6A–D). CD8 + EMRA cells, linked to both senescence and inflammation^83,84^, were more frequent in lymphoma patients, possibly contributing to their compensatory immune responses^24^. Despite these differences, lymphoma patients successfully generated durable vaccine-induced memory T cells that were comparable to those of HCs.

### Lymphoma patients have augmented antigen-specific Th1 and reduced cTfh cells.

We next investigated antigen-specific CD4 + T cell subsets, including Th1, Th2, Th17, and circulating T follicular helper (cTfh) cells, using markers CXCR3, CXCR5, CCR4, and CCR6. While total Th1 cells were the dominant population in both lymphoma patients and HCs (Fig. 6E, F, Extended Fig. 8A, B), antigen-specific Th1 and Th2 populations were significantly higher in lymphoma patients, compared to HC (p = 0.0074, p = 0.0011, respectively). This suggests that the expanded Th1 population may contribute to compensatory CD8 + T cell priming in patients with impaired humoral immunity (Fig. 6E). Th17 and Th1/Th17 cell populations were significantly lower in lymphoma patients compared to HC, (p = 0.0057 and p = 0.0011). There was a numerical but not significant trend towards reduced antigen-specific cTfh cells in lymphoma patients (p = 0.178) (Fig. 6F). This may be explained by impaired germinal center B cell formation, which also potentially affects cTfh cell response^85^.

Previous studies on MS patients^5–18^ and hematologic cancer patients^86^ treated with aCD20 and infected with COVID-19 disease have suggested that compensatory T cell mechanisms might develop in response to B cell depletion, facilitating antiviral responses in these patients. Our findings indicate that lymphoma patients capable of mounting T cell responses compensate for impaired B cell activity by producing antigen-specific CD8 + T cells comparable to HC but, through elevated CD8 + EMRA cells.

### aCD20-treated lymphoma patients exhibited altered antigen specific Treg and Th1 populations.

Next, we used unsupervised clustering to examine the differentiation states of vaccine-induced, antigen-specific memory T cells across treatment groups (Fig. 6G, H, Extended Fig. 8C, D, Extended Fig. 9A, B). Th1 cells in cluster 3 were more frequent in lymphoma patients, with the highest levels in those treated with aCD20 ± CT (Fig. 6G, H). Conversely, Treg cells in cluster 2 are less frequent in lymphoma patients with aCD20 ± CT treatment. Cluster 14, representing CD4 + EM1 cells with Foxp3 (CD25 + and CD127+) expression, was more frequent in aCD20 treated compared to HC (Fig. 6G, H). In aCD20 + CT− treated patients, the activated antigen-specific cell frequency in cluster 8 with high expression of HLA-DR, T-bet, ICOS, CD38^24,86^, and CXCR3 (low) was slightly elevated compared to HC (Fig. 6G, H), and cluster 8 weakly correlates with IFNg (r = 0.2, p = 0.002), suggesting that when B cell immunity is compromised, there can be increased T cell induction (Extended Fig. 8D).

As in traditional gating, CD8 + EMRA cells in clusters 12 (GrzmB+) and 13, showed higher frequencies in lymphoma patients, especially in the aCD20 ± CT-treated group (Fig. 6G, H).

Given the limitations of the AIM assay for capturing all antigen-specific T cells, particularly CD8 + T cells^33,87^, we utilized markers (CD38, HLA-DR, PD1, ICOS, and GzmB) to examine the antigen-specific CD8 + T cell activation^33^. Cluster 7, comprising activated EM1-like CD8 + cells, showed a trend towards increased abundance in aCD20 + CT patients than in HCs (Fig. 6.G, H). This cluster strongly correlated with non-naive antigen-specific CD8 + T cells (r = 0.8, p < 0.0001) and weekly correlates with IFNγ (Extended Fig. 8D).

In summary, lymphoma patients treated with B-cell depleting therapies, who were able to mount a vaccine-induced T cell response exhibited a decreased frequency of regulatory cells and an increased frequency of Th1 and CD8 + EMRA cells within the antigens-specific memory T cells compartment. These findings suggest a compensatory shift toward T cell-driven immunity in the context of impaired B cell function. Studies in animal models and early clinical trials have demonstrated that depleting regulatory T cells using anti-CD25 antibody can enhance vaccine-induced T cell responses and improve vaccine efficacy against viruses^88^.

### Lack of T cell response (IFNg) to vaccination correlates with cancer relapse and survival

In a related observation, all four patients who ultimately succumbed to illness prior to the one-year follow-up exhibited minimal or no IFNg response to SARS-CoV-2 vaccination (Extended Fig. 10A). Notably, 60% of patients (3 out of 5) who relapsed from lymphoma during this period failed to generate vaccine-induced IFNγ responses (Extended Fig. 10A, B). These findings strongly suggest that impaired functional immunity, evidenced by inadequate SARS-CoV-2-specific T cell responses, may underlie lymphoma specific immune surveillance and serve as a surrogate marker for overall prognosis and lymphoma-related survival.

## Discussion

Most existing studies on SARS-CoV-2 vaccine efficacy in cancer patients have focused on heterogenous cohorts of multiple cancer subtypes undergoing various treatments^20 45^. In this study, we investigated how lymphoma histology and treatment regimen- including aCD20, aCD20 + CT, CT alone, or monitoring-impacted vaccine-induced immune responses. While prior studies in hematologic malignancy patients have primarily assessed humoral responses to vaccination, in this study we performed paired analysis of both T and B cell responses and identified immune predictors of vaccine efficacy. Our data demonstrates that lymphoma patients exhibit attenuated antibody responses and impaired T cell immunity, with the most profound deficits in those treated with aCD20 + CT. Using high-dimensional flow cytometry, we generated detailed T and B cell atlases, including patients with markedly depleted B cell populations. The phenotypic differences in global T and B cell subsets post vaccination were shaped by both the malignancy and treatment regimens, with the overall impact of SARS-CoV-2 vaccination on the bulk population remaining below 1%. We further identified phenotypic features within T and B cells compartments that predicted both antibody and T cell responses and subsequently analyzed SARS-CoV-2 vaccine specific memory phenotypes in these populations.

Anti-spike IgG levels in lymphoma patients were strongly influenced by treatment type. Patients receiving aCD20 ± CT exhibited markedly lower antibody levels. However, some patients in the aCD20-treated groups retained the ability to generate antibody responses, and notably, most patients in the CT treatment group retained the capacity to produce antibody responses comparable to HC, despite reduced B cell counts. Importantly, these patients also displayed an intact CD4 + T cell profile, suggesting that CD4 + T cells might support residual B cell function and facilitate antibody production^86,89^. Antibody response correlated with specific B cell subsets, including naïve, transitional, classical memory, and DN2 B cells, that may partially escape depletion during lymphoma-directed therapy. Conversely, DN3 cells were negatively associated with antibody response. DN3 cells have previously been characterized as exhausted and dysfunctional and are linked to disease progression and poor treatment responses in patients with head and neck squamous carcinoma (HNSCC) and melanoma^90^. In evaluating T-B cell coordination, we found that the correlation between IFNγ-production and IgG was significantly reduced in aCD20 ± CT treated patients. In contrast, CT-treated and MON group patients demonstrated a moderate correlation, underscoring the reliance of T and B cell cooperation.

Despite compromised humoral immunity, lymphoma patients who mounted effective T cell responses exhibited SARS-CoV-2 antigen-specific T cell levels comparable to those of HC. Moreover, similar to healthy controls^91,92^, these patients demonstrated spike-specific memory T cells distributed across durable memory types (CM, EM, and EMRA). Our findings suggest that lymphoma patients with sufficient T cell numbers maintain a functional and robust T cell-directed antiviral response capable of polyclonal T cell expansion following vaccination. Additionally, late and end-stage differentiation phenotypes (EM3, EMRA), critical for T cell durability, recirculation, tissue access, and responses upon antigen re-exposure, were observed at a higher frequency in lymphoma patients. Both EM and EMRA play pivotal roles in antiviral immunity^93,94^, and in the context of SARS-CoV-2 vaccination, the presence of activated antigen-specific EM and EMRA is indicative of a strong and effective vaccine response in healthy individuals^33^.

In aCD20 ± CT treated patients, we observed a decreased frequency of vaccine-specific Tregs compared to HC. Tregs cells play a critical dual role in modulating the immune response following SARS-CoV-2 vaccination^95^. While essential for preventing immunopathology by controlling excessive immune activation, their suppressive activity can also attenuate the magnitude or durability of vaccine-induced protective immunity^96,97^. The reduced frequency of T regulatory cells in aCD20 ± CT-treated patients was accompanied by an increased frequency of vaccine-specific Th1^88^ and more differentiated EMRA CD8 + T cells relative to HC. B cell depletion may diminish regulatory B cell activity, thereby relieving suppression on CD8 + T cell responses^25,98,99^. It is also possible that impaired antibody response may result in higher antigen levels, potentially driving CD8 T cells differentiate towards an EMRA phenotype. CD8 + T cells play a critical role in viral clearance^33,100^, even when humoral immunity is impaired^24,33,74,82,101–104^. Patients with hematologic malignancies and MS on aCD20-based therapies frequently exhibit compensatory CD8 + T cell responses following COVID-19 infection^86,105^. In aCD20 ± CT-treated patients, the observed expansion of vaccine-specific Th1 cells alongside increased frequency of EMRA CD8 + T cells likely reflect a compensatory T cell-mediated immune response in the context of B cell-depletion. Moreover, effective humoral immunity requires B cell-cTfh cell interactions^68,106^, as impaired virus-specific cTfh cell has been linked to severe COVID-19^107^. Consistent with this, our data revealed slightly reduced frequencies of cTfh cell in T cell-responsive lymphoma patients compared to healthy controls.

We identified significant correlations between naïve CD4 + and CD8 + T cells, transitional, naïve, classical memory, and DN2 B cell subsets with the magnitude of anti-spike antibody responses. Conversely, activated CD8 + EM3 and EMRA T cell frequencies in bulk CD3 T cells negatively correlated with anti-spike antibody levels across samples. These findings suggest a balanced interplay or “crosstalk between B and T cell responses is critical to prevent breakthrough infections in vaccinated immunocompromised patients. At one-year post-vaccination, most lymphoma patients experiencing progression or death showed compromised SARS-CoV-2 vaccine responses, predominately characterized by deficient T immunity (Extended Fig. 10A, B).

Consistent with previous preclinical models and clinical studies^71^, our data underscore the multifaceted impact of aCD20 treatment on both B and T cell immunity following SARS-CoV-2 vaccination. Nonetheless, patients treated with anti-CD20 therapies may still achieve T cell-driven protection against COVID-19, despite impaired humoral responses. Notably, patients with preserved T cell compartments, such as those in CT treatment group appear capable of mounting effective anti-viral responses even in the context of compromised B cell immunity.

Although comprehensive, our study has some limitations. Lymphoma patients represent a highly vulnerable population prioritized for early SARS-CoV-2 mRNA vaccination. As a result, the collection of matched pre-vaccination samples was not possible in all cases. Within lymphoma, our sample also was heterogeneous; patients were of different histologies, disease stages, and at varying treatment time points and treatment regimens. There was also variability between the time between last treatment and vaccination. While all patients received treatment within six months prior to vaccination, the degree of B cell depletion post-infusion varied individually, and no standardized cutoff exist for defining B cell recovery after treatment^25^. Consequently, heterogeneity in timing from infusion or treatment cycle may have minimally influenced the immune patterns observed. Furthermore, limited T cell numbers in some patients restricted our ability to phenotype the antigen-specific T cell population comprehensively. Although variation trends in SARS-CoV-2 spike antigen-specific T cell responses were noted across lymphoma treatment groups compared to healthy controls, the small sample sizes limited statistical power to detect significant differences in some cases. While parts of the bulk T and B cell phenotype analyses remain descriptive, these data provide valuable insights given the inherent heterogeneity of patient cohorts, encompassing diverse lymphoma subtypes and treatments that are collectively analyzed in prior studies.

Our well-characterized, treatment-specific cohorts provide a critical framework for dissecting the complex immunological impact of lymphoma and its therapies on T and B cell immunity. Vaccination in lymphoma patients with a perturbed immune system offers valuable insights into the relative contributions of different arms of the immune system to protective immunity, which is also pertinent to other populations receiving aCD20 therapy^108^ or chemotherapy. Understanding vaccine immunogenicity in lymphoma patients is essential, as their unique immune landscapes might influence responses to vaccines against various pathogens, including seasonal influenza. Furthermore, the observation that patients treated with aCD20 ± CT had reduced SARS-Cov-2 vaccine-specific regulatory T cells has important implications for cancer vaccine strategies. Notably, multiple clinical trials have investigated anti-CD25 monoclonal antibody therapies aimed at depleting Tregs to potentially enhance cytotoxic CD8 + T cell responses^88,109,110^. These findings have broad implications for vaccine strategy development in immunocompromised individuals and underscore the potential for cancer vaccines to elicit effective immune protection.

## Methods

### Patient Characteristics and Study Design

To monitor the induction of antigen-specific immune responses to SARS-CoV-2 vaccination, we collected both serum and peripheral blood mononuclear cells (PBMCs) from 50 lymphoma (LM) patients and 10 healthy controls (HC) (Table 1.1 and 1.2) 2–4 weeks after the, second dose of the Pfizer-BioNTech or Moderna SARS-CoV-2 vaccine. Of the 50 lymphoma patients, 11 received no treatment (MON), 12 underwent anti-CD20 antibody therapy (Ritixumab) (aCD20), 22 underwent anti-CD20 therapy plus chemotherapy (aCD20+CT), and 6 had chemotherapy alone (CT) (Fig. 1A). Of the 50 lymphoma patients, 14 had follicular Lymphoma (FL), 8 had Marginal zone lymphoma (MZL), 13 had diffuse large B cell lymphoma (DLBCL), 5 had classical Hodgkin Lymphoma (HL), 1 had lymphocyte predominant Hodgkin lymphoma (LPHL), and 4 had Waldenstrom Macroglobulinemia (WM) (Extended fig 1A) All treatments were administered before receiving the SARS-CoV-2 vaccine. Although antibody and ELISpot analyses were performed on all patients, PBMCs from 42 lymphoma patients were used in the flow cytometry study due to insufficient cell counts in the 10 other patients. Following sample isolation, PBMCs were immediately cryopreserved. PBMCs were later thawed one day before the initiation of experimental assays. We included 11 pre-vaccinated lymphoma patients’ samples and 10 pre-vaccinated healthy controls (matched sample to the post-vaccinated healthy controls) (Tables 1.2 and 1.3). Healthy control samples were provided by the NYU vaccine center.

### Ethics

All study activities were performed under an approved protocol (number S20–02069) of the NYU IRB, and each participant provided written informed consent.

### Serological Analyses

A patient’s serologic status towards SARS-CoV-2 was assessed using the following method (**Multiplex Bead-Binding Assay**):

Antibody profiling was conducted using a multiplex bead-binding assay as previously described^26,111^. Biotinylated SARS-CoV-2 antigens-including spike and receptor-binding domain (RBD) proteins produced in-house, as well as nucleocapsid protein (Sino Biological 40588-V27B-B)-were immobilized on streptavidin-coated QBeads (Sartorius, 90792). Each bead population, distinguished by unique internal fluorescence, was coupled to a specific antigen to allow simultaneous detection of multiple antibody specificities. Heat-inactivated plasma samples were diluted in PBS with 0.1 % Tween 20 and 1% skim milk (Millipore Sigma, 1.15363.0500) and incubated with the antigen-coated beads. Bound antibodies of IgG, IgA, and IgM isotypes were detected using fluorophore-conjugated secondary antibodies specific for each isotype (Jackson Immunoresearch: anti-human IgG-Alexa 488 (J109–545-098), anti-human IgA-PE (J109–115-011) and anti-human IgM-DyLight405 (J 709–475-073)). Following incubation and washing steps, samples were analyzed by flow cytometry using a Yeti ZE5 Cell Analyzer (Bio-Rad). Data was processed with FlowJo software (version 10.7.1). For reference standards, a commercially available human monoclonal antibody clone, CR3022 in the IgG, IgA and IgM formats, originally isolated from SARS-CoV02 infected individuals (Absolute antibody Human IgG1, Kappa, Ab01680–10.0, Human IgA, Kappa, Ab01680–16.0, Human IgM, Kappa, Ab01680–15.0) were included in triplicates in each measurement. To standardize the data across different measurements, reference MFI values of the control antibodies, CR3022, was used. Positivity thresholds were defined based on median fluorescence intensities (MFI) from pre-pandemic 6 negative control samples, calculated as the mean plus three standard deviations as previously described^112^ (healthy adult serum samples were from the NYU Center for Biospecimen Research & Development). Evidence of past SARS-CoV-2 infections (MBI seropositive) was defined conservatively based on anti-spike total protein and spike domain responses in a subject’s pre-vaccination samples that were >3SD over mean levels in pre-pandemic healthy controls.

IgG responders were defined as those with IgG levels greater than 1000 U(MFI), low responder status was defined as IgG levels between 400–999 U(MFI), while non-responders were defined by an IgG antibody level of less than 400 U(MFI).

### SARS-CoV-2 Spike Protein Specific Peptide Mega Pool

To detect T cell reactivity against the SARS-CoV-2 spike antigen (in ELISpot and flow cytometry AIM assays), we stimulated PBMCs from patients and HCs using a spike-specific peptide mega pool covering the entire spike glycoprotein antigen (15-mer each) with an overlapping region of 11 residues (Miltenyi Biotec PepTivator^®^ SARS-CoV-2 Prot S Complete, cat 130–129-712), as previously described^57,113^.

### T cell Activation

Cryopreserved PBMCs were thawed and stimulated using a commercially available SARS-CoV-2 spike peptide library as previously described^74^, at a final concentration of 1 μg/ml. Briefly, PBMCs were thawed and washed in 10 ml of warmed complete medium (RPMI supplemented with 10% FCS, 2 mM of L-glutamine, 100 U ml^−1^ of penicillin). For stimulation, cells were placed in 96-well plates with 200 μL of fresh RPMI and peptide mega pool for 24 hours in a humidified incubator at 37°C and 5% CO_2_. Matched unstimulated samples for each donor were rested overnight in the incubator.

### IFNγ ELISpot Assay for the Detection of Cytokine-producing T cells

For the evaluation of functional T cell responses to SARS-CoV-2-specific antigens, the IFNγ enzyme-linked immunospot (ELISpot) assay was used, as previously described^114^. Briefly, 200,000 freshly thawed PBMCs from patients and healthy controls were stimulated *in vitro* with 1 μg of spike peptide pool/ml (15-mer peptide PepTivator libraries (Miltenyi Biotec) for 24 hours. The number of activated T cells was determined using the *ImmunoSpot* IFNγ kit (Cellular Technology Limited) according to the manufacturer’s instructions, and spots were counted using a CTL S6 EM2 ELISpot reader (Immunospot). To quantify SARS-CoV-2-specific responses, mean spots of the control wells were subtracted from the positive wells, and the results were expressed as spot-forming units (sfu) per 10^6^ PBMCs. Responses were considered positive if the results were at least three times the mean of the negative control wells and >18 SFU per 10^6^ PBMCs.

### Antibodies

Flow antibodies (fluorophore, target, clone, manufacturer) used in the phenotype analysis are presented in Extended Table 1.

### Detection of SARS-CoV-2-specific T cells and Bulk Immune Cell Phenotyping by Flow Cytometry

SARS-CoV-2-specific T cells were detected using an activation-induced marker (AIM) assay, which also contains markers to identify several T and B cell phenotypes (Extended Table 2). Unstimulated (“baseline”) and peptide-stimulated PBMCs were analyzed simultaneously using a 35-color panel (Table S1) optimized for the Cytek Aurora spectral flow cytometry instrument. Both peptide-stimulated and unstimulated PBMCs (~1 × 10^6^ cells) were centrifuged and washed in FACS buffer (HBSS + 2% FBS). PBMCs were resuspended in FACS buffer with 5 μl of Fc receptor block (BioLegend, no. 422302) and monocyte block (BioLegend, no. 422302) and incubated for 5 min at 22 °C. To prevent Fc block from interfering with IgG staining, anti-IgG antibody in 20 μL FACS buffer was preincubated for 5 minutes at 22 °C before the addition of the blocking buffer. Subsequently, 25 μl of antibody cocktail (Table S1) was added, and samples were incubated for 30 min at 22 °C. Cells were washed in HBSS twice and resuspended in Zombie UV fixable viability dye (BioLegend, no. 423106) diluted in HBSS. Cells were washed in HBSS buffer, fixed, and permeabilized for 30 minutes at room temperature per the manufacturer’s instructions using the Foxp3 Fixation/Permeabilization buffer set (eBioscience, 00–5523-00). Cells were then washed twice in 1X permeabilization buffer before staining for intracellular antibodies diluted in permeabilization buffer for 1 hour at 4 °C. Cells were then washed in permeabilization buffer twice and resuspended in FACS buffer before data acquisition. Cells were analyzed on a 5-laser Cytek Aurora spectral flow cytometer using Cytek SpectroFlo software. The FCS files were analyzed in FLOWJO (BD, version 10.9).

### High-Dimensional Analysis and Statistics

Up to 1 × 10^6^ cells were analyzed using FlowJo v10 (Treestar) software. Gating strategies are illustrated in extended figures Extended Fig.1G, Extended Fig.4A, B, and Extended Fig. 5A UMAP analyses were conducted on single, live, and CD3+ T cells or CD19+ B cells or spike antigen-specific T cells. Flow cytometry data in the form of CSV files containing marker intensities exported from FlowJo were first converted into FCS files using a customized R script. Data for samples were merged based on phenotypes using analytic functions available in flowCore^115^ and CATALYST^116^. Batch correction was performed using the Combat program^117,118^ to remove any potential variance caused by different experiment dates. 100,000 events were randomly selected (equal down-sampling of 100,000 cells from each FCS file) for dimension reduction and cluster identification using the Louvain algorithm^119,120^, for computational efficiency. The uniform manifold approximation and projection (UMAP)^121^ was used for clustering visualization. Several clustering resolutions were evaluated, and the best clustering resolution was selected and manually assigned phenotypes using heatmaps (Tables 3,4, and 5). All statistics were calculated using the Wilcoxon test^122,123^ function available in R, and plotted using the ggplot2 package (v3.4.1). Lymphoma patients in the UMAP clusters were grouped by treatment cohorts, IFNg responders (R) and non-responders (NR), antibody responders (IgG-R; high response, IgG-LR; low response), and non-responders (IgG-NR). There are some patients whose level of IFNg or IgG was not measured, and those patients were not included in the UMAP cluster histograms.

### Spearman Correlations

Cluster frequencies and clinical parameters for each patient and healthy control were correlated in R (v4.2.2) using Spearman with the function *corrplot* v0.92. Cluster frequencies were used to calculate principal components using the function *prcomp* from the package stats (v4.2.2) and first normalizing the input data so that mean and variance were zero for each parameter before PCA. Next, principal components 1 and 2 were plotted using *ggplot2* (v3.4.1). NA values were omitted from the analysis.

### Statistical Analysis

Multiple comparisons across different cohorts were done with the Kruskal-Wallis test and post hoc Dunn’s test^124^ in Prism (version 9.1.2, GraphPad software) unless otherwise specified. Statistical pairwise comparisons were performed using the nonparametric Mann-Whitney test in Prism. Statistical pairwise comparisons between matched samples were performed using the Wilcoxon test in Prism. P values were adjusted for multiple comparisons when appropriate in Prism. All tests were performed in a two-sided Mann Whitney test using a nominal significance threshold of P<0.05 unless otherwise specified. Correlation coefficients between two parameters were quantified by Spearman’s rank correlation coefficient with nonparametric methods (null hypothesis r=0) to assess the corresponding statistical significance. *p<0.05; **p<0.01; ****p<0.0001; ns - not significant.

## Supplementary Files

This is a list of supplementary files associated with this preprint. Click to download.

- table1.tif
- extendedtable1.jpg
- extendedtable2.jpg
- extendedtable3.jpg
- extendedtable4.jpg
- extendedtable5.jpg
- extendedtable6.jpg
- extendedFig.1.jpg
- Extendedfig.2.jpg
- extendedFig.3.jpg
- extendedFig.4.jpg
- extendedFig.5.jpg
- extendedFig.6.jpg
- extendedFig.7.jpg
- extendedFig.8.jpg
- extendedFig.9.jpg
- extendedFig.10.jpg
- GA.png
